# Supplementary material for: Extrinsic Regulators of mRNA Translation in Developing Brain: Story of WNTs
Source: Cells. 2021 Jan 28;10(2):253. doi: 10.3390/cells10020253 (PMC7911671; doi:10.3390/cells10020253)
Supplement: Supplementary file 1 [file cells-10-00253-s001.pdf]

Supplementary Figures:

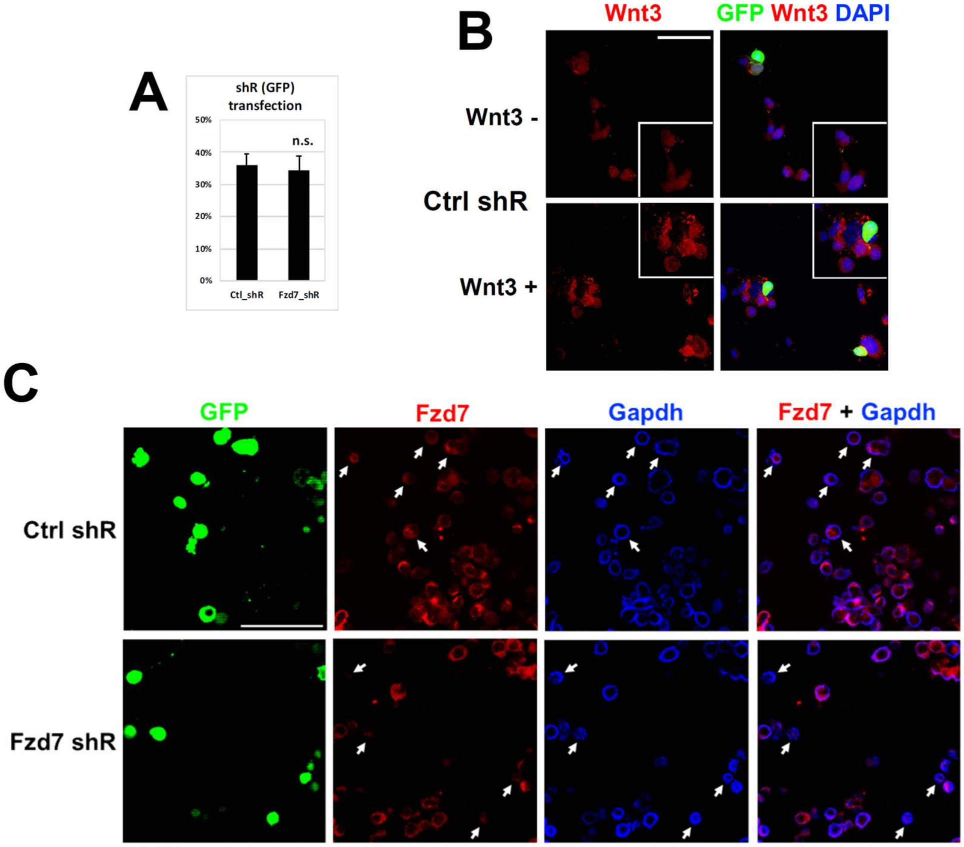

Figure S1. (**A**). Transfection efficiency of shRNA plasmids into N2a cells with counting GFP-transfected cells (n = 19~ 26 repeat percentages with total 2,099 ~ 2,243 GFP cells). Data represent the mean and SEM. n.s.: not significant. (**B**). IHC for Wnt3 (red), GFP (green), and DAPI (blue) in N2a cells after 3 days of *Ctrl* shRNA transfection containing GFP marker and following 4 hours of either Wnt3– (mock: nothing added) or Wnt3+ (100 ng/ml) treatment (n = 3 separate transfections). Insert: higher magnification image; scale bar: 100 μm. (**C**). IHC for GFP (green), Fzd7 (red), and Gapdh (blue) in N2a cells after 3 days of each shRNA transfection containing GFP marker (*Ctrl* or *Fzd7* shRNA) and following 4 hours of Wnt3 (100 ng/ml) treatment (n = 3 separate transfections). Arrow: shRNA-transfected GFP cells; scale bar: 100 μm.

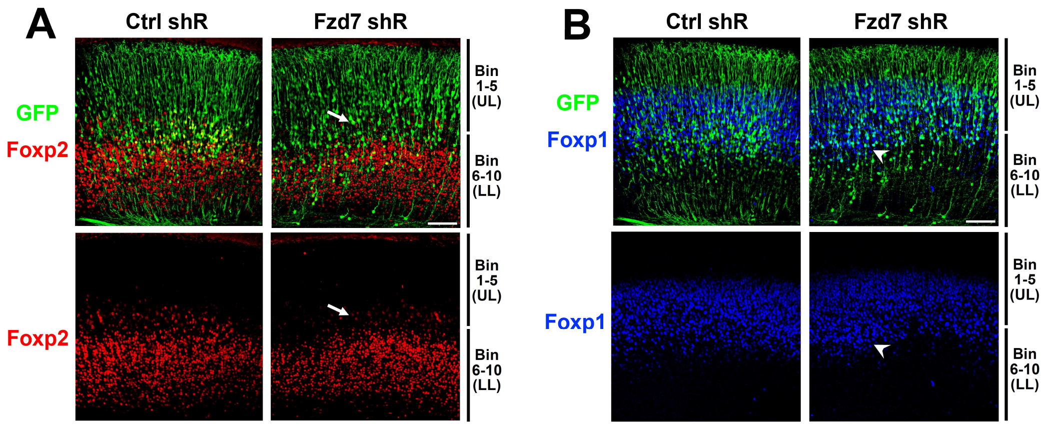

Figure S2. (**A**)–(**B**). IHC for GFP (green), Foxp2 (red in **A**), and Foxp1 (blue in **B**) in P0 neocortices electroporated *in utero* with *Ctrl* or *Fzd7* shRNA at E13 (n = 3 animals). Arrow and arrowhead: the areas showing bigger change in Foxp2 and Foxp1 expression, respectively; Bin 1-5: upper layers (UL); Bin 6–10: lower layers (LL); scale bar: 100 μm.

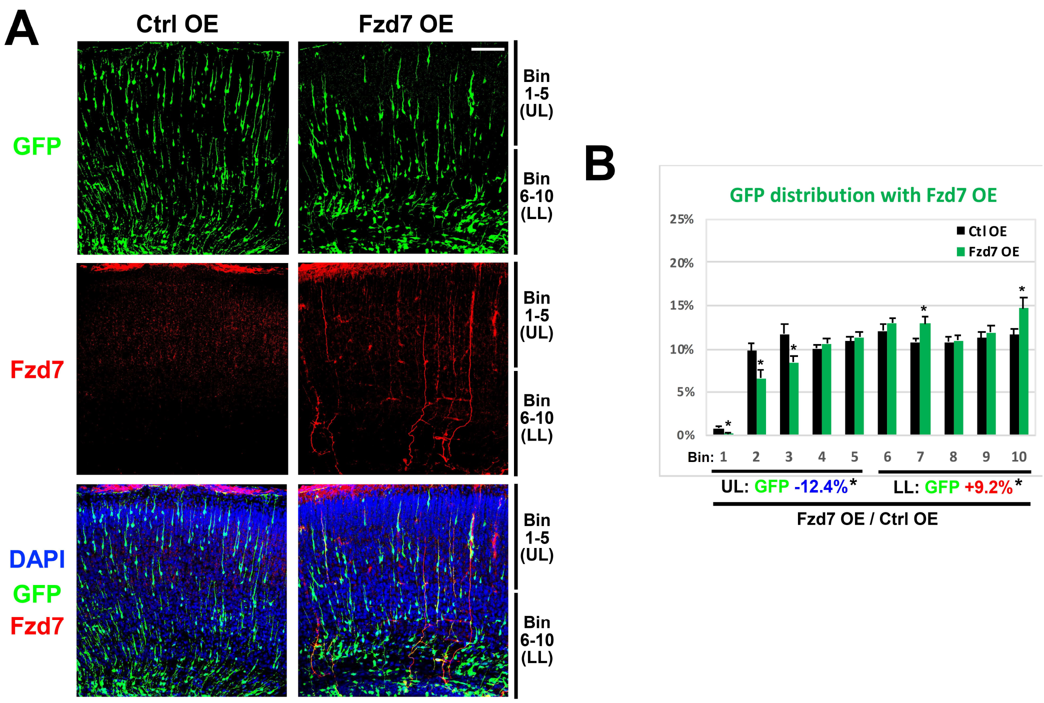

Figure S3. (**A**). IHC for GFP cells (green) with Fzd7 (red) immunostaining in P0 neocortices electroporated *in utero* with *Ctrl* or *Fzd7* OE at E16 ($n = 3$ animals). DAPI shown in blue; Bin 1–5: upper layers (UL); Bin 6-10: lower layers (LL); scale bar: 100 μm. (**B**). Percentage (%) of GFP+ cells in each bin from total 10 bins (n = 21 ~ 25 pictures counted with total ~ 4,000 GFP cells). GFP+ cells in the upper layers (Bin 1–5) of *Fzd7* OE neocortices decreased by 12.4% compared to *Ctrl* OE, while increase of 9.2% was shown in the lower layers (Bin 6–10). Data represent the mean and SEM. *: $p < 0.05$.

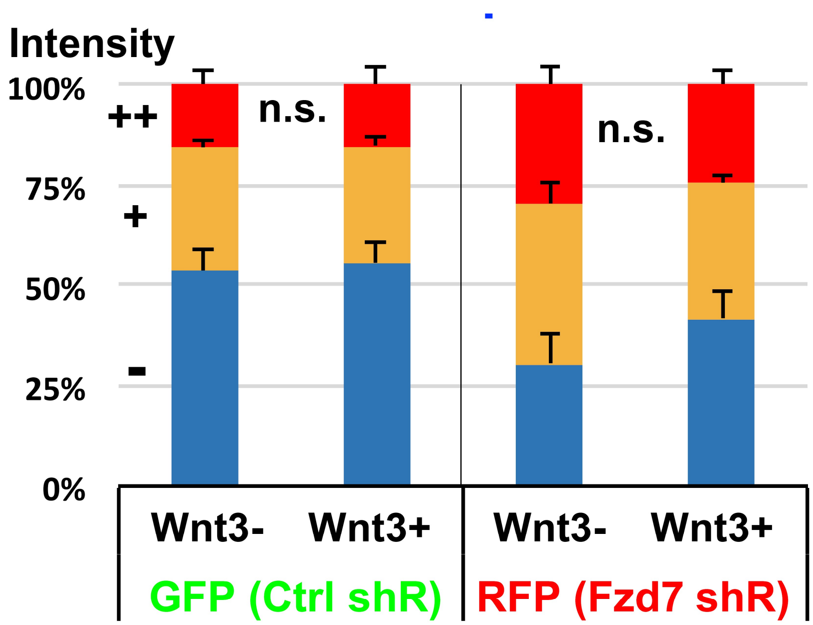

Figure S4. Quantification of Foxp1 signal in GFP (*Ctrl* shRNA) and RFP (*Fzd7* shRNA) cells with 48 hours treatment of Wnt3- or + (100 ng/ml) after six days *in vitro* (DIV) cultures of primary neuronal cells *in utero* electroporated (IUE) at. E13 (n = 3 animals). The -, +, and ++ intensities were normalized to the intensity observed in non-transfected cell as an intrinsic control of immunostaining quality (n = 20 ~ 32 pictures counted with total 297 ~ 490 cells). Data represent the mean and SEM. n.s.: not significant.
